# Effects of feeding different levels of dietary corn silage on growth performance, rumen fermentation and bacterial community of post-weaning dairy calves

**DOI:** 10.5713/ab.23.0174

**Published:** 2023-10-26

**Authors:** Lingyan Li, Jiachen Qu, Huan Zhu, Yuqin Liu, Jianhao Wu, Guang Shao, Xianchao Guan, Yongli Qu

**Affiliations:** 1 College of Animal Science and Veterinary Medicine, Heilongjiang Bayi Agricultural University, Daqing 163319, China; 2 Key Laboratory of Low-carbon Green Agriculture in Northeastern China, Ministry of Agriculture and Rural Affairs P. R. China, Heilongjiang Bayi Agricultural University, Daqing 163319, China; 3 Bright Farming Co., Ltd, Shanghai 201103, China; 4 Heilongjiang Academy of Agricultural Sciences, Qiqihaer 161006, China

**Keywords:** Corn Silage, Growth, Post-weaning Dairy Calves, Rumen Bacterial Community, Rumen Fermentation

## Abstract

**Objective:**

The objective of this study was to evaluate the growth performance, rumen fermentation parameters and bacterial community of post-weaning dairy calves in response to five diets varying in corn silage (CS) inclusion.

**Methods:**

A total of forty Holstein weaned bull calves (80±3 days of age;128.2±5.03 kg at study initiation) were randomized into five groups (8 calves/group) with each receiving one of five dietary treatments offered as total mixed ration in a 123-d feeding study. Dietary treatments were control diet (CON; 0% CS dry matter [DM]); Treatment 1 (T1; 27.2% CS DM); Treatment 2 (T2; 46.5% CS DM); Treatment 3 (T3; 54.8% CS DM); and Treatment 4 (T4; 67.2% CS DM) with all diets balanced for similar protein and energy concentration.

**Results:**

Results showed that calves offered CS had greater average daily gain, body length and chest depth growth, meanwhile altered rumen fermentation indicated by decreased rumen acetate concentrations. Principal coordinate analysis showed the rumen bacterial community structure was affected by varying CS inclusion diets. *Bacteroidetes* and *Firmicutes* were the predominant bacterial phyla in the calf rumens across all treatments. At the genus level, the abundance of *Bacteroidales_RF16_group* was increased, whereas *Unclassified_Lachnospiraceae* was decreased for calves fed CS. Furthermore, Spearman’s correlation test between the rumen bacteria and rumen fermentation parameters indicated that *Bacteroidales_RF16_group* and *Unclassified Lachnospiraceae* were positively correlated with propionate and acetate, respectively.

**Conclusion:**

The results of the current study suggested that diet CS inclusion was beneficial for post-weaning dairy calf growth, with 27.2% to 46.5% CS of diet DM recommended to achieve improved growth performance. *Bacteroidales_RF16_group* and *Unclassified Lachnospiraceae* play an important role in the rumen fermentation pattern for post-weaning calves fed CS.

## INTRODUCTION

When managing dairy calves, much emphasis has traditionally been directed toward feeding and management protocols from birth through weaning whilst they are being fed milk [1,2]. However, feeding and management programs at and shortly after weaning are also critical to the subsequent health and growth performance for growing calves. Post-weaning calves still have a developing rumen and associated rumen fermentation [3]. Digestive capacity and ability to use nutrients are still limited compared to older heifers which may affect the use of fibrous forages. Therefore, ensuring sufficient physical and metabolic function development of the rumen will benefit optimum body weight (BW) gains [4].

Dietary forage can improve rumen development and health for post-weaning calves, which has been positively correlated with rumen motility, stimulating rumination, rumen size, and microbial development [5,6]. Typically, alfalfa or oat hays have been used in supplementation of grain starters by dairy producers of China to feed early post-weaning calves. Compared with the alfalfa or oat hay, some dairy farms which have less resources and labor would like to use corn silage (CS) to feed calves due to its availability on-farm and lower cost. In addition, CS is a palatable and nutritious forage containing higher starch and moisture concentrations than dry hay products. Beiranvand et al [7] reported that the addition of water to dry calf starter, up to 50% moisture, led to increased total volatile fatty acids (VFA) production, dry matter intake (DMI), and average daily gain (ADG). However, the feeding of CS to young calves is still controversial. Some studies found calves consuming CS had greater growth, with increased protein digestibility and final rumen mucosal weights [8,9]. On the other hand, early post-weaning calves have limited rumen capacity and DMI may be limited with higher fiber and moisture diets, which may impair rumen papillae development, and decrease BW and dry matter digestibility [10,11]. Kehoe et al [12] found that calves fed CS reduced rumen and intestinal measurements, which may reduce efficiency in nutrient utilization.

Rumen bacteria, which are the most abundant and diverse ruminal microbes, play critical roles in fermenting plant proteins and polysaccharides to generate the nutrients necessary for maintenance and growth [13,14]. However, a wide range of metabolites that perform a vast array of complex metabolic activities in the rumen are also used by the microbes for their own proliferation [15]. The rumen microbiome can be dramatically influenced by changes in feeding strategies and diets [16,17]. Limited research has been conducted in evaluating the effects of dietary CS level on the rumen bacterial community of post-weaning calves. Therefore, the objective of this study was to investigate the effects of providing different levels of CS on growth performance, rumen fermentation parameters and bacterial community of post-weaning calves.

## MATERIALS AND METHODS

### Animal ethics statement

The animal care and experimental procedures were approved by the Animal Welfare and Ethics Committee of Heilongjiang Bayi Agriculture University (No.202106). Animal care and handling were followed the guidelines by the regulations for the Administration of Affairs Concerning Experimental Animals (The State Science and Technology Commission of China, 1988).

### Corn silage management

Corn (*Zea mays* L.; Pioneer hybrid 335; Pioneer Hi-Bred International Inc. Beijing, China) used in this study was planted in May 2018 and harvested at the two-third milkline stage of maturity in mid-September in the central region of Heilongjiang (Yuda farm; Suihua, Heilongjiang, China). Whole-plant corn was directly harvested using a forage harvester (John Deere model 8400; John Deere Agricultural Machinery Co., Ltd, Harbin, China) with knives adjusted to a 1.7-cm length of cut. Forage was ensiled in bunker silos and covered with black plastic and tires for 70 d prior to the start of experiment. Nutrient composition of the CS and Oat hay are presented in Table 1.

### Animals and feeding

Forty Holstein weaned bull calves (80±3 days of age) at the dairy farm of Heilongjiang Academy of Agricultural Sciences (Suihua, China) with initial BW of 128.2±5.03 kg were selected and randomly divided into five groups with eight calves per group. Five diets, (Table 2), were fed to the groups as follows: control diet (CON), containing 0% CS DM; Treatment 1 (T1), containing 27.2% CS DM; Treatment 2 (T2), containing 46.5% CS DM; Treatment 3 (T3), containing 54.8% CS DM; and Treatment 4 (T4), containing 67.2% CS DM. Diets were formulated using CPM-Dairy software (version 3.0.8.01; Cornell University, Ithaca, NY; University of Pennsylvania, Kennett Square, PA; and William H. Miner Agricultural Research Institute, Chazy, NY; [CPM]) and balanced to achieve similar protein concentrations (mean = 17.2% CP) and energy densities (mean = 2.38 Mcal ME/kg DM). Calves were housed individually and the 5 experimental diets were offered as total mixed ration twice per day at 0600 and 1700 h to provide 5% orts/d based on the intake from the previous day. The experiment feeding period lasted for 123 d and the first ten days were taken as adaption period. After no abnormal behaviors were observed during the adaptation period, then initial data such as BW and feed intake were recorded. Water was offered for *ad libitum* consumption, and feed intake was weighed every 2 wk to calculate the average DMI during the experiment phase. Corn silage and diets were collected monthly, subsampled, and submitted for nutrient analysis.

### Sample collection

Calves in different treatment groups were all weighed before the morning feeding for 3 consecutive days at the beginning and end of the experiment to calculate the initial BW, final BW, and ADG. In addition, body measurements, including body length, withers height, chest girth and chest depth were taken at the beginning and end of the trial.

Rumen contents of six calves per treatment were collected for three hours after morning feeding in the end of the experiment via esophageal tubing as described by Paz et al [18]. by the same technician with the same procedure. A portable pH meter (Testo 205; Testo AG, Schwarzwald, Germany) was used to determine the pH of ruminal fluid immediately following collection. The ruminal content samples were divided into 2 parts. The first part was immediately stored in liquid nitrogen for microbial DNA extraction. The second part was filtered through 4 layers of sterile cheesecloth, then centrifuged at 3,000×g for 20 min and taking the supernatant. Then, 1 mL of supernatant was mixed with 0.25 mL of metaphosphoric acid standard solution (250 g/L) for the determination of NH_3_-N and VFA.

### Rumen fluid NH**_3_**-N and volatile fatty acids analysis

Rumen fluid NH_3_-N was analyzed using a spectrophotometer (UV-1700; Shimazu Corporation, Kyoto, Japan) following the methods described by Broderick and Kang [19]. The VFA concentrations were determined by gas chromatograph (GC-2014; Shimazu Corporation, Japan) with a capillary column (Rtx-Wax, 30 m long, 0.25 mm diameter, 0.25 μm film). Chromatograph parameters included an injection volume of 0.4 μL with a split ratio of 40:1, column nitrogen flow rate of 2.5 mL/min, injector temperature of 220°C and flame ionization detector temperature of 250°C. The oven temperature program was as follows: initial 110°C for 30 s, up to 120°C at 10°C/min, 120°C hold for 4 min, and continue to 150°C at 10°C/min.

### Rumen Bacteria DNA extraction, polymerase chain reaction amplification and sequencing

Bacterial DNA was extracted using E.Z.N.A. Bacterial DNA Kit (Omega Bio-tek, Norcross, GA, USA) following the manufacturer’s instruction. The bacterial cell wall was removed by lysozyme digestion, followed by Proteinase K digestion. Following lysis, binding conditions were adjusted and the sample was applied to a HiBind” DNA spin-column. Two rapid wash steps removed trace salts and protein contaminants, and finally DNA was eluted in water or low ionic strength buffer. Purity and quality of the genomic DNA were evaluated on 1.2% agarose gels, and the quantity of the DNA was detected by a NanoDrop NC2000 spectrophotometer (Thermo Fisher Scientific, Waltham, MA, USA) and then stored at −80°C until being used as a template for the polymerase chain reaction (PCR) assays.

Barcoded primers 338F (5′-ACTCCTACGGGAGGCAG CA-3′) and 806R (5′-GGACTACNNGGGTATCTAAT-3′) were used to amplify the V3–V4 region of the bacterial 16S rRNA gene. Sample-specific 7-bp barcodes were incorporated into the primers for multiplex sequencing. A well-optimized 25 μL reaction system was carried out as follows: 5 μL 5×Reaction Buffer, 5 μL 5×GC Buffer, 2 μL (2.5 mM) dNTP, 1 μL (10 μM) of each Forward and Reverse primers, 2 μL DNA template, 8.75 μL ddH_2_O, and 0.25 μL Q5 DNA Polymerase (NEB, Ipswich, MA, USA). PCR amplification consist of an initial denaturing step at 98°C for 2 min followed by 27 cycles of 98°C for 15 s, annealing at 50°C for 30 s, and extension at 72°C for 30 s with a final extension at 72°C for 5 min. PCR amplicons were purified with Vazyme VAHTSTM DNA Clean Beads (Vazyme, Nanjing, China) and quantified using the Quant-iT PicoGreen dsDNA Assay Kit (Invitrogen, Carlsbad, CA, USA). After the individual quantification step, amplicons were pooled in equal amounts, and pair-end 2×250 bp sequencing was conducted using the Illumina NovaSeq platform with NovaSeq 6000 SP Reagent Kit at Shanghai Personal Biotechnology Co., Ltd (Shanghai, China).

### Bioinformatic analysis

The raw sequence data were analyzed by using QIIME2 2019.4. Then, sequences were quality filtered, denoised, merged and chimera removed using the DADA2 plugin. Non-singleton amplicon sequence variants (ASVs) were aligned with mafft and used to construct a phylogeny with fasttree2. Alpha-diversity metrics and beta diversity metrics were estimated using the diversity plugin with samples rarefied to 54821sequences per sample. Taxonomy was assigned to ASVs using the classify-sklearn naïve Bayes taxonomy classifier in feature-classifier plugin against the SILVA Release 132 Database. ASV-level alpha diversity indices including Chao1 richness estimator, Simpson index, Shannon diversity index and Observed species were calculated using the ASV table in QIIME2. Principal coordinate analysis (PCoA) and permutational multivariate analysis of variance (PERMANOVA) results with 999 permutations performed based on unweighted-unifrac distance metrics using R software (V4.0.3). Correlation analyses between rumen fermentation parameters and bacterial community (relative abundance >1%) were calculated using Spearman’s correlation test, and connections with p<0.05 and r>0.5 were retained.

Co-occurrence networks were constructed to identify the main rumen bacteria of strongly associated ASVs. We utilized the trimmed means of M (TMM) normalized counts per million (CPM) counts with the function “BioConductor” in the “edgeR” package and conducted Spearman’s correlation test between ASVs by using the package “Hmisc”, significant positive correlation was defined by ρ>0.7 and p<0.001. All networks were visualized with the Fruchterman-Reingold layout with 10^4^ permutations in igraph. The packages “sciplot” and “ggmisc” were used to visualize the abundance of intergroup difference modules in a network.

### Statistical analysis

For feed intake and DMI were analyzed using PROC MIXED of SAS version 9.4 (SAS Institute Inc., Cary, NC, USA) with sample time (day) as repeated measures during the overall experiment. The statistical model was: Y_ijk_ = μ+T_i_+D_j_+T_i_×D_j_+ C_k_+ɛ_ijk_; where Y_ijk_ was the observed variable, μ was the overall mean, T_i_ was the fixed effect of diet treatment, D_j_ = repeated measure of sample day, T_i_×D_j_ is the interaction between treatment and sample day, C_k_ was the random effect of calves, and ɛ_ijk_ denotes the residual error.

Body weight, ADG, Feed efficiency, body measurements, rumen fermentation parameters and relative abundance of rumen bacteria data were determined using the general linear model (GLM) of SAS. The statistical model was: Y_ij_ = μ+T_i_+ɛ_ij_; where Y_ij_ was the observed variable, μ was the overall mean, T_i_ was the fixed effect of diet treatment, ɛ_ij_ denotes the residual error. Least squares means estimates were reported. For all statistical analyses, significance was declared at p<0.05 and trends were determined at 0.05<p<0.10. When a significant effect of treatment was detected, differences between the means were tested using the Bonferroni multiple comparison test.

## RESULTS AND DISCUSSION

### Growth performance

The growth performance results are summarised in Table 3. The initial BW was not different among dietary treatments. Calves fed diets containing 27.2% (T1) and 46.5% (T2) CS DM had higher ADG and final BW than these fed diets containing 0 (CON), 54.8% (T3), and 67.2% (T4) CS DM (p< 0.05). The feed efficiency (ADG/DMI) were not different among different treatment groups. Daily gain is often of primary consideration when discussing the development of post-weaning dairy calves. In the current study, ADG results were close to targets (0.8 to 1.0 kg/d) recommended for Holstein heifers [20,21]. We found ADG and final BW were increased for calves offered less than 50% CS in the diet; however, ADG and final BW were decreased when CS exceed 50% DM in the diet. This may be explained by the lower DMI for calves fed higher CS diet. Because calves have limited rumen capacity and DMI may be limited with high moisture diets. Furthermore, Akins [4] reported that post-weaning nutrition to maintain higher gains of 0.9 kg/d can reduce days until first breeding. Our study indicates that a diet with 27.2% to 46.5% CS DM would allow for post-weaned calves to reach a desired growth rate and attain an earlier breeding age.

### Body measurements

The results of calf body measurements are shown in Table 4. Body measurements including body length, withers height, chest girth and chest depth were not different at the beginning of the experiment. Greater body length and chest depth growth were observed for calves offered CS compared with CON diet (p<0.05).

Calf growth parameters are useful indicators of future dairy cow performance [22]. In the present study, calves assigned to the CS treatment groups exhibited greater body length and chest depth growth, however chest girth were not different in the body measurements. Body length and chest depth are mainly determined by bone growth, which are an early maturing part of the body while chest girth has been more highly correlated with meat and fat deposition [23]. Kehoe et al [12] found that chest girth and withers height growth were not different for calves fed different levels of CS diets. Therefore, calves which are fed diets containing CS had better skeletal growth rather than adipose tissues development.

### Rumen fermentation

The effects of different levels of dietary CS on rumen fermentation are presented in Table 5. Ruminal pH, NH_3_-N, propionate and butyrate concentrations did not differ among the treatments. Calves fed diets with more than 50% CS had lower acetate concentration compared to those fed CON diet.

In the present study, ruminal pH ranged from 6.12 to 6.39, which fell into the optimum range for microorganism growth and without risk for rumen acidosis [24]. The NH_3_-N concentration is affected by ruminally degradable protein and the supply of energy in the rumen [25,26]. In the current study, we observed no significant differences in NH_3_-N concentration of dietary treatment groups. Moreover, we found calves fed CS diets had decreased acetate concentration compared to those fed oat hay. Consistent with our results, Beiranvand et al [27] and Chen et al [28] found provision of alfalfa or oat hay had increased acetate but did not affect butyrate production for post-weaning calves at 70 d of age. Zou et al [29] found ruminal butyrate concentration was lower for post-weaning calves at 95 d of age when offered CS compared with alfalfa or oat hay. Kehoe et al [12] indicated that feeding solely CS as starter feed stunted the growth of rumen papillae, however no significant differences were found for growth, gain, or health of calves at 8 wk of age. These results showed that calves fed higher dietary CS level could lower the acetate concentration in the rumen.

### Alpha and beta diversity analysis

A total of 2,440,087 clean tags were obtained after the rigorous quality control from 30 samples, with an average of 81,336 for each sample and the average read length is 363 bp. Rarefaction analysis was conducted to assess ASV coverage, producing a Good’s coverage value >0.98 for each sample, implying that the sequence coverage was sufficient. Indices of alpha diversity here included Chao1 richness estimator, Simpson index, Shannon diversity index and Observed species are shown in Table 6. No differences were observed for calves fed CS compared with control group.

**Figure 1:**
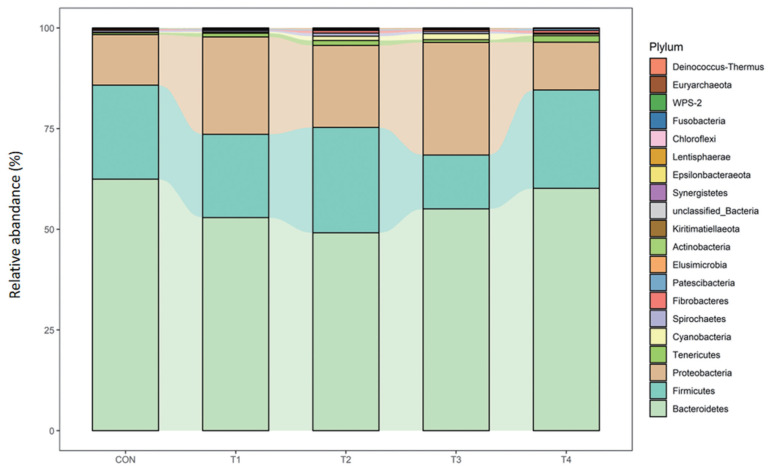
Ruminal plyla (Relative Abundance of the top 20) composition of calves fed different dietary corn silage.

We then used a PCoA to examine the influence of dietary CS levels on rumen bacterial structure of calves. As shown in Figure 1, there was an obvious confidence ellipses separation for bacterial communities of different treatment groups. The confidence ellipses for calves fed different CS level were partially overlapped, while confidence ellipses of T2, T3, and T4 were completely separated from CON. Furthermore, PERMANOVA also showed that the effects of feeding CS on the structure of the bacterial communities was significant (p = 0.004).

### Structure of dominant rumen bacterial communities

**Figure 2:**
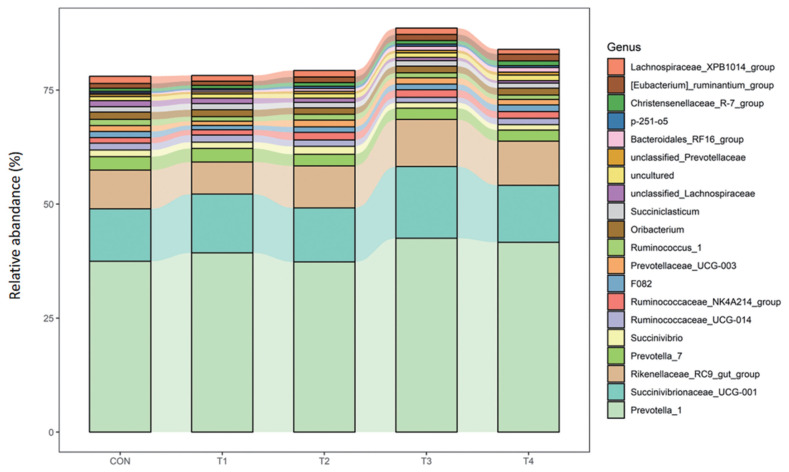
Ruminal Genus (Relative Abundance top 20) composition of calves fed different dietary corn silage.

At the phylum level, *Bacteroidetes* (49.13% to 62.48%) followed by *Firmicutes* (13.41% to 26.16%) and *Proteobacteria* (11.86% to 27.96%) were the most three abundant of all groups, accounting for more than 95% of the phyla. No difference was observed for calves fed dietary CS compared with control group (Table 7; Figure 2).

**Figure 3:**
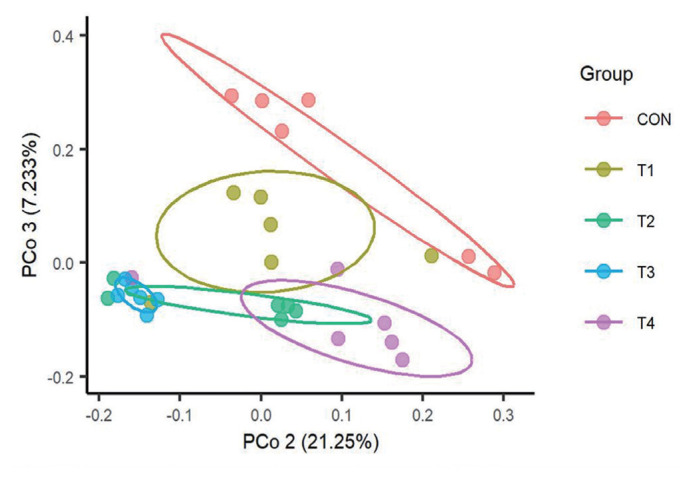
Principal coordinate analysis (PCoA) of rumen bacteria among the five treatment groups based on unweighted unifrac distance matrix.

At the genus level, *Prevotella_1*, *Succinivibrionaceae_UCG-001*, and *Rikenellaceae_RC9_gut_group* were the most abundance of all the treatment groups, accounting for more than 50% of the genera. The abundance of *Bacteroidales_RF16_group* was significantly higher for calves fed CS compared to those fed CON diet (p<0.01). Compared with calves fed CON, calves offered CS had lower abundance of *Unclassified_Lachnospiraceae* (p<0.05) (Table 8; Figure 3).

In this study, consistent with the view that the rumen microbial ecosystem is dominated by a core community composed of microbes [16], the dominant phyla were Bacteroidetes and Firmicutes, followed by *Proteobacteria* which plays a great role in the formation of biofilms and digestion of soluble carbohydrates.

Variations at the genus level provided a deep insight into the profile of bacterial community composition in response to diet. In the present study, *Prevotella_1*, *Succinivibrionaceae_UCG-001*, and *Rikenellaceae _RC9_gut_group* were the most abundant three ones of all the groups. A previous study showed that species of *Prevotella* grow rapidly when fermentable carbohydrates are available [30]. *Succinivibrionaceae* has been reported to produce succinate, a precursor for propionate [31] and valerate synthesis by other microbes in the rumen. Hernandez-Sanabria [32] found that the relative abundance of *Succinivibrio* of rumen bacteria was 8.45% to 12.49% for cattle when the fed the diet with ME of 2.6 Mcal/kg. The results of our study was consistent with it. *Rikenellaceae _RC9_gut_group* was also reported to produce succinate and propionate as fermentation end products [33]. Thus, these findings suggested that bacteria genera associated with the synthesis of propionate had become the core community in the rumen.

*Bacteroidales_RF16_group* was found to be higher and *Unclassified_Lachnospiraceae* was lower for calves fed CS in the current study. Other studies have demonstrated the *Bacteroidales RF16 group* was positively correlated with propionate and negatively correlated with isobutyrate [34,35]. *Unclassified Lachnospiraceae* are not only abundant members of the rumen microbial community, but also play a very important role in fermenting pectin and had positive correlations with ADG [36–38] . However, the mechanism of *RF16* and *Unclassified Lachnospiraceae* family metabolism is not yet clear.

### Correlation analysis

**Figure 4:**
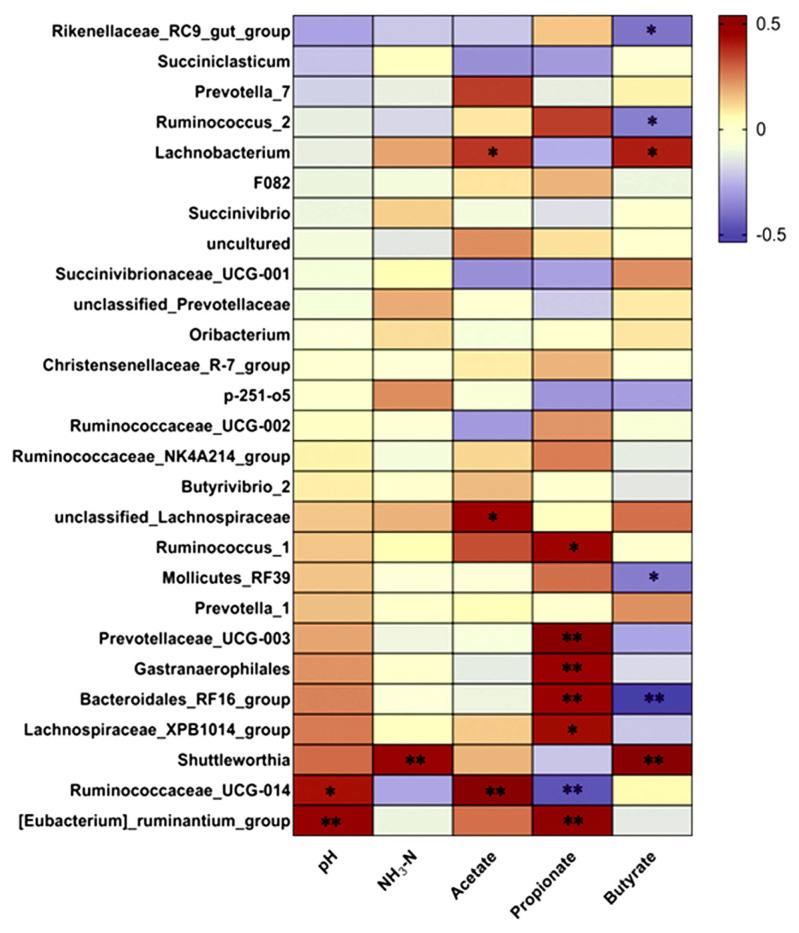
Heatmap of the correlations between rumen fermentation parameters and genus abundance. The genera representing at least 1% of the bacterial community are presented. Lattices are colored based on the corresponding Spearman’s correlation test. * p<0.05; ** p<0.01.

A correlation analysis (Figure 4) was conducted to evaluate the genus relationship with rumen fermentation parameters. The relative abundances of *Ruminococcaceae_UCG-014* (r = 0.421, p = 0.021) and *[Eubacterium]_ruminantium_group* (r = 0.487, p = 0.006) were positively correlated with pH value. The relative abundance of *Shuttleworthia* (r = 0.286, p = 0.008) was positively correlated with NH_3_-N. The relative abundances of *Lachnobacterium* (r = 0.363, p = 0.048), *unclassified_Lachnospiraceae* (r = 0.449, p = 0.013) and *Ruminococcaceae_UCG-014* (r = 0.540, p = 0.002) were positively correlated with acetate. *Prevotellaceae_UCG-003* (r = 0.538, p = 0.002), *Gastranaerophilales* (r = 0.469, p = 0.009), *Bacteroidales_RF16_group* (r = 0.469, p = 0.009) and *[Eubacterium]_ruminantium_group* (r = 0.502, p = 0.005) were positively correlated with propionate, whereas the relative abundance of *Ruminococcaceae_UCG-014* (r = −0.464, p = 0.002) was negatively correlated with propionate. The relative abundances of *Lachnobacterium* (r = 0.399, p = 0.029) and *Shuttleworthia* (r = 0.532, p = 0.002) were positively correlated with butyrate, but the relative abundances of *Rikenellaceae_RC9_gut_group* (r = −0.396, p = 0.03), *Ruminococcus_2* (r = −0.373, p = 0.042), *Mollicutes_RF39* (r = −0.381, p = 0.038) and *Bacteroidales_RF16_group* (r = −0.535, p = 0.002) were negatively correlated with butyrate.

Dietary composition plays a predominant role in determining both the rumen bacteria community and the metabolic function of the rumen [39]. In this study, *Bacteroidales_RF16_group* and *Unclassified Lachnospiraceae* were positively correlated with propionate and acetate respectively. Meanwhile, the abundance of *Bacteroidales_RF16_group* was increased and *Unclassified Lachnospiraceae* was decreased in the CS diets treatment, and we observed increased propionate and decreased acetate concentration for calves fed CS. These results suggested CS diet altered rumen bacterial community and promoted the growth of rumen bacteria enabling to produce propionate rather than acetate. The presence of the *Bacteroidales_RF16_group* and *Unclassified Lachnospiraceae* play a key role in rumen fermentation pattern for calves fed CS diets.

### Rumen bacterial co-occurrence patterns

**Figure 5:**
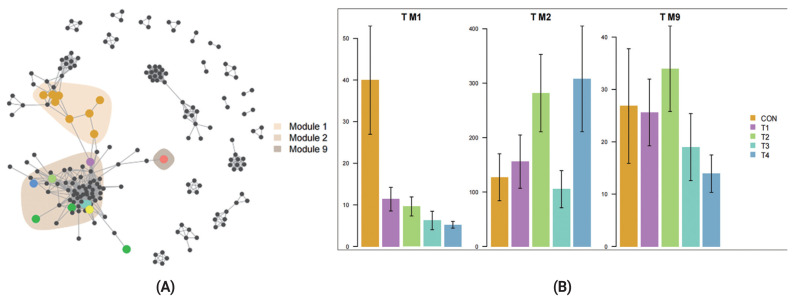
(A) The co-occurrence network visualizing significant positive correlations (ρ>0.7 and p<0.001, shown with gray lines) among bacteria amplicon sequence variants (ASVs) of different treatment groups. ASVs abundant among different groups were colored in dark yellow for CON, purple for T1, green for T2, pale blue for T3, and dark blue for T4. The insensitive ASVs that are displayed in gray. Shaded areas indicate that the modules contain ASVs in the top 10 most populated network modules. (B) Cumulative relative abundance (as counts per million, CPM; y-axis in ×1,000) of ASVs in the 3 treatment-sensitive modules. Error bars indicate standard error of the mean. T M1 = treatment module 1; T M2 = treatment module 2; T M9 = treatment module 9.

We also investigated how different dietary CS levels impacted co-occurrence patterns of rumen bacterial communities and explored the distribution trend of ASVs in co-occurrence patterns. We found ASVs assigned to CON diet were predominantly located in Module 1 (M1) and separated from module 2 (M2) and module 9 (M9) in which ASVs were affiliated with the CS diet groups (Figure 5A). M1 containing primarily CON ASVs, M2, and M9 containing primarily T4 and T2 ASVs respectively (Figure 5B). The results demonstrated that different dietary CS levels presented an important driver in rumen bacterial communities for post-weaning calves.

Co-occurrence network analysis is widely applied to explore the connections in microbial communities [40]. Bacterial taxa that extensively and frequently interact with other taxa in the co-occurrence network are thought to play critical roles and core members in the microbiome [40,41] . In our study, we found that the ASVs specific to different groups clustered into distinct modules. ASVs of CON diet were predominantly located in M1 and ASVs of CS diet were located in M2 and M9. Furthermore, our data showed that corn-silage feeding has an obvious (p = 0.004) influence on community β-diversity (Figure 3). The results revealed that feeding different levels of CS had greatly influenced microbial community structure.

## CONCLUSION

Results from this study show that calves fed diets containing moderate levels of CS (27.2% to 46.5% of DM) had greater ADG, body length and chest depth growth, while having decreased acetate concentration compared with CON. Principal coordinate analysis and co-occurrence network analysis showed different CS diets significantly altered bacterial community structure of rumen. At the genus level, the abundance of *Bacteroidales_RF16_group*, which was positively correlated with propionate, was increased, whereas *Unclassified_Lachnospiraceae*, which was was positively correlated with acetate, was decreased. In addition, it appears that *Bacteroidales_RF16_group* and *Unclassified Lachnospiraceae* play an important role in the rumen fermentation pattern of post-weaning calves.

## Figures and Tables

**Table 1:** Nutrient composition of whole plant corn silage and oat hay (DM %)

Item	Nutrient							

	DM	CP	NDF	ADF	Ash	Starch	Ca	P
Corn silage	30.5	9.10	39.4	23.3	5.53	31.7	0.20	0.34
Oat hay	89.1	13.3	55.7	33.9	12.6	1.20	0.63	0.31

DM, dry matter; CP, crude protein; NDF, neutral detergent fiber; ADF, acid detergent fiber.

**Table 2:** Dietary ingredient and nutrient concentrations of treatment diets

Item	Diet^1)^				

	CON	T1	T2	T3	T4
Ingredient (% of DM)					
Oat hay	14.0	0	0	0	0
Corn silage	0	27.2	46.5	54.8	67.2
Steam flaked corn	30.1	23.2	7.68	3.22	0
Soybean meal (44% CP)	12.8	21.2	21.7	24.7	23.5
Whole linted cottonseed	11.0	5.94	5.04	3.00	0
Soybean hulls	13.4	6.82	5.04	3.00	0
Dried distillers grains	10.0	6.24	4.72	0.00	0
Sodium chloride	0.72	0.74	0.70	0.70	0.70
Di-calcium phosphate	0.46	1.16	1.16	1.38	1.30
Limestone	1.60	1.53	1.47	1.31	1.31
Sodium bicarbonate	1.00	1.00	1.00	1.00	1.00
Vitamin premix^2)^	5.00	5.00	5.00	5.00	5.00
Nutrient compositions (% of DM)					
DM, % as fed	90.21	74.31	63.19	58.27	50.79
CP	17.35	17.74	17.50	17.30	16.33
NDF	29.35	24.39	28.99	28.43	29.59
ADF	18.48	14.87	17.93	17.78	17.91
Starch	19.77	23.49	20.51	21.44	22.13
Ca	0.86	0.92	0.90	0.90	0.87
P	0.45	0.49	0.45	0.45	0.40
Energy estimate^3)^					
ME (Mcal/kg)	2.47	2.49	2.35	2.33	2.26
NEm (Mcal/kg)	1.70	1.72	1.66	1.64	1.60
NEg (Mcal/kg)	1.21	1.21	1.09	1.10	1.03

DM, dry matter; CP, crude protein; NDF, neutral detergent fiber; ADF, acid detergent fiber; ME, metabolise energy; NEm, net energy for maintenance; NEg, net energy for growth.

1) CON, control diet (0% CS DM); T1, Treatment 1 (27.2% CS DM); T2, Treatment 2 (46.5% CS DM); T3, Treatment 3 (54.8% CS DM); T4, Treatment 4 (67.2% CS DM).

2) The premix provided the following per kilogram of the diet: Vitamin A, 1,500 IU; Vitamin D, 200 IU; Vitamin E, 400 IU; Fe, 72.5 mg, Zn, 100 mg; Mn, 60 mg; Cu, 12.5 mg; Se, 1.0 mg; Co, 0.5mg.

3) Calculated according to NRC (2001).

**Table 3:** Effects of different dietary corn silage levels on growth performance of calves

Item	Treatment^1)^					SEM	p-value

	CON	T1	T2	T3	T4		
Initial BW (kg)	136.6	136.6	130.9	142.8	136.4	9.59	0.78
Final BW (kg)	220.5^a^	255.3^b^	247.5^b^	230.5^ab^	222.5^a^	17.27	0.021
Feed intake (kg/d)	6.63	9.99	11.52	10.20	11.90	2.34	0.22
DMI (kg/d)	5.98	6.77	6.52	5.95	6.05	1.41	0.97
ADG (kg/d)	0.74^a^	1.05^b^	0.98^bc^	0.78^ac^	0.76^ac^	0.10	0.047
Feed efficiency (ADG/DMI)	0.13	0.15	0.15	0.13	0.13	0.04	0.91

SEM, standard error of the mean; BW, body weight; DMI, dry matter intake; ADG, average daily gain.

1) CON, control diet (0% CS DM); T1, Treatment 1 (27.2% CS DM); T2, Treatment 2 (46.5% CS DM); T3, Treatment 3 (54.8% CS DM); T4, Treatment 4 (67.2% CS DM).

a–c Mean values within a row with different superscript letter differ significantly (p<0.05).

**Table 4:** Effects of different dietary corn silage levels on body measurements of calves

Item	Treatment^1)^					SEM	p-value

	CON	T1	T2	T3	T4		
Initial							
Body length (cm)	95.5	91.3	95.1	93.3	90.7	4.46	0.74
Withers height (cm)	96.4	97.0	95.7	98.3	97.1	2.41	0.90
Chest girth (cm)	119.6	119.3	118.1	121.2	119.8	2.54	0.58
Chest depth (cm)	51.9	50.5	47.5	48.5	47.1	2.41	0.27
Final							
Body length (cm)	111.8^a^	116.3^ab^	126.3^b^	116.7^ab^	119.3^ab^	3.95	0.024
Withers height (cm)	114.3	115.3	115.8	113.7	116.7	3.46	0.91
Chest girth (cm)	143.3	150.7	147.0	148.3	145.3	4.44	0.55
Chest depth (cm)	58.7	60.0	60.2	62.7	62.0	1.43	0.097
Growth							
Body length (cm)	16.2^a^	25.0^bc^	31.2^c^	23.3^ab^	28.6^bc^	3.71	0.013
Withers height (cm)	17.9	18.3	20.1	15.4	19.6	1.80	0.13
Chest girth (cm)	23.7	31.4	28.9	26.2	25.5	3.20	0.20
Chest depth (cm)	6.80^a^	9.47^ab^	12.70^bc^	14.20^c^	14.90^c^	2.00	0.009

SEM, standard error of the mean.

1) CON, control diet (0% CS DM); T1, Treatment 1 (27.2% CS DM); T2, Treatment 2 (46.5% CS DM); T3, Treatment 3 (54.8% CS DM); T4, Treatment 4 (67.2% CS DM).

a–c Mean values within a row with different superscript letter differ significantly (p<0.05)

**Table 5:** Effect of different dietary corn silage levels on rumen fermentation parameters of calves

Item	Treatment^1)^					SEM	p-value

	CON	T1	T2	T3	T4		
pH	6.17	6.39	6.33	6.20	6.12	0.33	0.071
NH_3_-N (mg/dL)	14.5	14.6	14.0	14.3	14.7	1.05	0.090
Acetate (mmol/L)	47.7^a^	44.8^ab^	43.5^ab^	42.3^b^	41.7^b^	1.34	0.004
Propionate (mmol/L)	23.6	23.8	26.5	25.6	26.8	0.44	0.063
Butyrate (mmol/L)	10.05	9.16	8.18	9.14	8.79	0.47	0.059

SEM, standard error of the mean.

1) CON, control diet (0% CS DM); T1, Treatment 1 (27.2% CS DM); T2, Treatment 2 (46.5% CS DM); T3, Treatment 3 (54.8% CS DM); T4, Treatment 4 (67.2% CS DM).

a,b Mean values within a row with different superscript letter differ significantly (p<0.05).

**Table 6:** Effects of different dietary corn silage levels on rumen bacterial alpha diversity indices of calves

Item	Treatment^1)^					SEM	p-value

	CON	T1	T2	T3	T4		
Chao1	2,113.9	2,924.4	3,014.8	2,717.0	2,211.0	628.4	0.50
Simpson	0.95	0.93	0.96	0.96	0.95	0.03	0.84
Shannon	7.15	7.41	8.11	7.30	7.26	0.90	0.83
Observed_species	1,891.8	2,482.0	2,715.4	2,292.9	1,945.1	537.1	0.51

SEM, standard error of the mean.

1) CON, control diet (0% CS DM); T1, Treatment 1 (27.2% CS DM); T2, Treatment 2 (46.5% CS DM); T3, Treatment 3 (54.8% CS DM); T4, Treatment 4 (67.2% CS DM).

**Table 7:** The proportion of dominant phylum in of calves fed different dietary corn silage

Item	Treatment^1)^					SEM	p-value

	CON	T1	T2	T3	T4		
Bacteroidetes	62.48	52.91	49.13	55.06	60.18	8.61	0.543
Firmicutes	23.33	20.68	26.16	13.41	24.43	6.25	0.306
Proteobacteria	12.54	24.19	20.38	27.96	11.86	9.13	0.333

SEM, standard error of the mean.

1) CON, control diet (0% CS DM); T1, Treatment 1 (27.2% CS DM); T2, Treatment 2 (46.5% CS DM); T3, Treatment 3 (54.8% CS DM); T4, Treatment 4 (67.2% CS DM).

**Table 8:** The top 20 bacteria with the highest relative abundance at the genus level

Item	Treatment^1)^					SEM	p-value

	CON	T1	T2	T3	T4		
Lachnospiraceae_XPB1014_group	1.58	1.24	1.41	1.38	1.05	0.19	0.119
[Eubacterium]_ruminantium_group	1.06	0.92	1.23	1.36	1.48	0.13	0.206
Christensenellaceae_R-7_group	0.66	0.71	0.82	0.78	1.04	0.09	0.075
p-251-o5	0.38	0.34	0.48	0.46	0.42	0.05	0.467
Bacteroidales_RF16_group	0.27^a^	0.49^b^	0.62^c^	0.89^d^	1.00^d^	0.07	0.008
unclassified_Prevotellaceae	0.51	0.44	0.51	0.56	0.64	0.06	0.443
uncultured	0.90	0.94	0.98	1.00	1.22	0.12	0.214
unclassified_Lachnospiraceae	1.31^a^	1.10^b^	0.93^b^	0.70^c^	0.55^d^	0.11	0.023
Succiniclasticum	1.17	1.37	1.19	1.22	1.11	0.14	0.844
Oribacterium	1.62	1.51	1.42	1.45	1.50	0.13	0.690
Ruminococcus_1	1.37	0.99	1.28	1.10	0.97	0.21	0.279
Prevotellaceae_UCG-003	1.29	0.93	1.49	1.39	1.21	0.16	0.234
F082	1.34	0.95	1.19	1.25	1.43	0.19	0.085
Ruminococcaceae_NK4A214_group	1.22	1.15	1.69	1.63	1.50	0.18	0.179
Ruminococcaceae_UCG-014	1.49	1.57	1.39	1.16	1.38	0.09	0.104
Succinivibrio	1.47	1.38	1.74	1.22	1.21	0.11	0.164
Prevotella_7	2.93	2.97	2.54	2.48	2.41	0.30	0.086
Rikenellaceae_RC9_gut_group	8.50	7.02	9.22	10.33	9.69	1.00	0.363
Succinivibrionaceae_UCG-001	11.50	12.90	11.84	15.72	12.49	1.35	0.274
Prevotella_1	37.47	39.31	37.32	42.52	41.63	4.51	0.436

SEM, standard error of the mean.

1) CON, control diet (0% CS DM); T1, Treatment 1 (27.2% CS DM); T2, Treatment 2 (46.5% CS DM); T3, Treatment 3 (54.8% CS DM); T4, Treatment 4 (67.2% CS DM).

a–d Mean values within a row with different superscript letter differ significantly (p<0.05).
